# Personalised 3D-Printed Mucoadhesive Gastroretentive Hydrophilic Matrices for Managing Overactive Bladder (OAB)

**DOI:** 10.3390/ph16030372

**Published:** 2023-02-28

**Authors:** Zara Khizer, Muhammad R. Akram, Muhammad Azam Tahir, Weidong Liu, Shan Lou, Barbara R. Conway, Muhammad Usman Ghori

**Affiliations:** 1 Department of Pharmacy, School of Applied Sciences, University of Huddersfield, Huddersfield HD1 3DH, UK; 2 College of Pharmacy, University of Sargodha, Sargodha 40100, Pakistan; 3 Institute of Pharmaceutical Technology and Biopharmaceutics, University of Bonn, 53113 Bonn, Germany; 4 Department of Pharmacy, Khalid Mahmood Institute of Medical Sciences, Sialkot 51310, Pakistan; 5 School of Computing and Engineering, University of Huddersfield, Huddersfield HD1 3DH, UK

**Keywords:** 3D printing, gabapentin, hydrophilic matrices, extended drug release, gastroretention, mucoadhesion, polyethylene oxide

## Abstract

Overactive bladder (OAB) is a symptomatic complex condition characterised by frequent urinary urgency, nocturia, and urinary incontinence with or without urgency. Gabapentin is an effective treatment for OAB, but its narrow absorption window is a concern, as it is preferentially absorbed from the upper small intestine, resulting in poor bioavailability. We aimed to develop an extended release, intragastric floating system to overcome this drawback. For this purpose, plasticiser-free filaments of PEO (polyethylene oxide) and the drug (gabapentin) were developed using hot melt extrusion. The filaments were extruded successfully with 98% drug loading, possessed good mechanical properties, and successfully produced printed tablets using fused deposition modelling (FDM). Tablets were printed with varying shell numbers and infill density to investigate their floating capacity. Among the seven matrix tablet formulations, F2 (2 shells, 0% infill) showed the highest floating time, i.e., more than 10 h. The drug release rates fell as the infill density and shell number increased. However, F2 was the best performing formulation in terms of floating and release and was chosen for in vivo (pharmacokinetic) studies. The pharmacokinetic findings exhibit improved gabapentin absorption compared to the control (oral solution). Overall, it can be concluded that 3D printing technology is an easy-to-use approach which demonstrated its benefits in developing medicines based on a mucoadhesive gastroretentive strategy, improving the absorption of gabapentin with potential for the improved management of OAB.

## 1. Introduction

Overactive bladder (OAB) is a symptom complex condition characterised by frequent urinary urgency, nocturia, and urinary incontinence with or without urgency [1,2]. It can affect people of any age and is the most common voiding dysfunction in the children [3]. The global prevalence of OAB in children is 15–20%, 10.8% for men and 12.8% for women that increases with age and severely affects the patients’ quality of life [4,5]. As per European and American studies, more than 10% of the population show symptoms and people older than 65 years are more likely to be affected [6]. Patients suffering from neurogenic diseases (multiple sclerosis, cerebral palsy, brain tumours, Parkinson’s diseases, cerebrovascular pathology, and spinal cord injuries) can develop symptoms of overactive bladder known as neurogenic detrusor overactivity or neurogenic OAB [7,8]. The neurogenic diseases affect the central nervous system responsible for controlling the functions and thus can cause neurogenic detrusor overactivity [9]. Contrarily, detrusor overactivity in non-neurogenic OAB can result from non-neurological diseases like urinary tract infection, muscle disease, bladder stones or can be idiopathic [2] or can be induced by drugs e.g. benzodiazepines and antidepressants [10,11,12]. 

The treatments available for OAB comprise oral pharmacotherapy medication, bladder training programs, modification in diet, surgery and electrical stimulation [10,13]. Anti-muscarinic drugs including oxybutynin, trospium chloride, solifenacin, darifenacin and tolterodine are 65–75% effective in reducing the OAB symptoms and are therefore, the first-line treatment for OAB [14,15,16]. Oral anti-muscarinic therapy for detrusor overactivity is usually prescribed for long period; nevertheless, the incidence of side effects associated with this therapy is relatively high. The most common side effects are due to blocking of muscarinic receptors in colon, salivary gland and ciliary smooth muscle induced constipation, dry mouth, and blurred vision, respectively. It also causes drowsiness and heart related side effects such as palpitations and arrhythmia that are difficult to tolerate for some patients, particularly the patients of older age who are likely to present cardiovascular comorbidities and the paediatrics [17,18,19] leading to poor patient compliance and adherence. For instance, as per discontinuation rate of anti-muscarinic drugs reported by Gopal et al., 42% of OAB patients discontinued the prescribed antimuscarinic medication after four months and the rate increased to 77% after 1 year [20]. In the UK clinical practice, repeat prescription data analysis reported a low adherence rate i.e. the average duration of using antimuscarinic drugs was only 3 months [21]. Furthermore, 30–40% of children suffering from OAB does not respond to anti-muscarinic treatment. In some neurogenic OAB cases, these drugs even fail to relieve the symptoms and lead to refractory neurogenic detrusor overactivity [22]. 

For many years, the researchers have targeted the detrusor muscle to depress the contractility via antimuscarinic drugs for the treatment of OAB [23]. However, the poor adherence, tolerability and patient compliance due to the bothersome side effects did not grant any success and hence the focus has shifted towards new interventions e.g. other mechanisms or structures of the bladder such as urothelial signalling and afferent nerves [11,24] which makes way for the centrally acting drugs in the treatment of OAB [25]. 

Gabapentin, a centrally acting anticonvulsant drug has not only used for the treatment of epilepsy but has also proven effective in psychiatric and neurologic disorders [26]. The mechanism of action of gabapentin is controversial and does not involve interaction with GABA (γ-aminobutyric acid) receptors [27]. It can cross the blood-brain barrier and is also used for sleep and anxiety disorders due to lack of toxicity. Gabapentin was used for the treatment of refractory interstitial cystitis, in the field of urology, for the first time [28,29]. The possible pathogenesis of interstitial cystitis in this regard was proposed as up-regulation of afferent C-fibre sensory neurons. The study reported reduced pain in 10 of 21 patients suffering from interstitial cystitis. The hypothesis was proposed that few cases of OAB show the same pathophysiology of up-regulation of afferent C-fibre sensory neurons which further promoted the use of gabapentin for the treatment of OAB [29]. Carbone et al., 2006 reported the effect of gabapentin on neurogenic overactive bladder. Gabapentin was effective in improving the urodynamic parameters and relieving the symptoms, in addition, no significant adverse effects were reported and none of the patient discontinued the treatment [7]. Similarly, Kim et al., 2004 investigated the effects of oral gabapentin in OAB patients who did not respond to the antimuscarinic therapy. It was reported that the drug was well-tolerated and symptoms were improved in 14 of 31 patients with refractory OAB [30]. 

However, when given orally, along with some favourable characteristics like absence of enzyme induction and hepatic metabolism and low protein binding [31,32] gabapentin has two major drawbacks i.e. short half-life (5–7 h) [33] and narrow absorption window as it is preferentially absorbed from upper small intestine [34,35,36] which in turn results in poor bioavailability [37] due to a saturable L-amino acid transport system. Owing to this, immediate release gabapentin requires frequent and multiple administration to achieve optimal efficacy. The multiple administration reduces the patient compliance, adherence and is unable to tolerate by some patients as gabapentin cause somnolence and dizziness as side effects [38,39]. Drugs with such drawbacks need a sophisticated and practical delivery system e.g., an extended release gastroretentive floating system. Floating systems are basically dynamically controlled systems with density lower than the gastric fluid (must be <1.004 g/cm^3^) that have enough buoyancy to float in the stomach for longer periods without affecting the gastric emptying rate and with desired drug release rate. The floating gastroretentive delivery system is suitable for drugs with narrow absorption window, short half-life, and low solubility at alkaline pH [40,41,42].

The rationale behind selecting this drug delivery system is to overcome the absorption-related and multiple administration drawbacks of gabapentin, allowing the dosage form to stay in the stomach for several hours along with the sustained release, thereby increasing the absorption extent. The development of gastroretentive floating drug delivery system where the dose can be easily modified and controlled is possible using additive manufacturing technology [43], i.e., the fused deposition modelling (FDM) technique, one of the most commonly and widely used additive manufacturing technologies [44]. The infill pattern and the number of shells are the basic parameters of FDM used to define the internal structure and outline the outer shape of the object to be printed. FDM can produce fully solid-filled structures and hollow objects by adjusting the infill density from 0% to 100%. The minimum number of shells required to print an object is one, however, the number of shells can be increased to enhance the strength and weight of the object as per the requirements, but this will take longer to print and more material is required.

In the present study, we aimed to address the absorption issue of gabapentin using hot-melt extrusion (HME) coupled with FDM without the aid of any plasticiser, while also facilitating the potential for personalised drug delivery. The literature [45,46] suggests some studies have developed gabapentin formulations; however, the question of personalisation remains unaddressed. Moreover, commercially these formulations are available as in fixed dose combinations or strengths, so the titration of dose according to the patient’s prerequisite is challenging to achieve. In current clinical practice, personalised medicines have become a reality which can go as far as to design drugs, medicines and devices which are tailored to individual patients’ pathophysiological needs, ensuring its acceptance by the body. The manufacturing of personalised drug delivery devices is a challenging process as it requires the manufacturing of small batches particularly tailored to the patient’s requirements which may not be economical and can be time consuming. Nonetheless, the recent emergence of 3D printing technologies has provided the potential for on-demand manufacturing, an easy path to the development of personalised drug delivery devices. In addition to any personalised aspect, the technology also provides an easy and efficient way of developing a gastroretentive floating drug delivery system to overcome the drawbacks of gabapentin. This research project is therefore aimed at overcoming the absorption-related issues of gabapentin by developing a gastroretentive floating drug delivery system via coupling HME and FDM. The developed filaments were used to fabricate a total of seven formulations with varying printing parameters, i.e., the infill density and shell number. The printed tablets were thoroughly characterised by performing geometric, ex vivo mucoadhesion, 3D surface texture analysis, in vitro floating and drug release study. Moreover, pharmacokinetic studies (in vivo drug absorption studies) were also carried out on the optimised formulation to evaluate the impact on drug absorption. 

## 2. Results and Discussion

### 2.1. Development and Characterisation of Filaments

**Figure 1:**
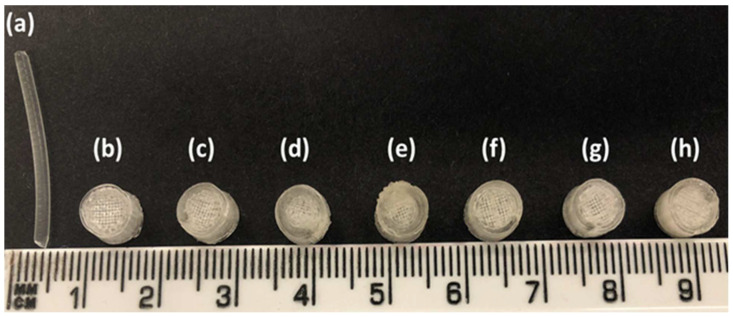
(**a**) Hot-melt extruded filament of gabapentin/polyethylene oxide (PEO); (**b**) F1 (1 shell with 0% infill); (**c**) F2 (2 shells with 0% infill); (**d**) F3 (3 shells with 0% infill); (**e**) F4 (4 shells with 0% infill); (**f**) F5 (2 shells with 10% infill); (**g**) F6 (2 shells with 20% infill); and (**h**) F7 (2 shells with 30% infill).

**Figure 2:**
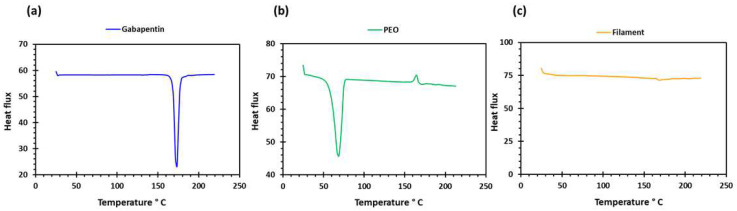
DSC profiles of (**a**) gabapentin; (**b**) polyethylene oxide (PEO); and (**c**) GBP/PEO filament.

**Figure 3:**
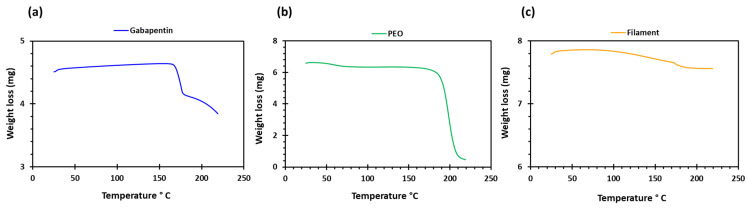
TGA profiles of (**a**) gabapentin; (**b**) polyethylene oxide (PEO); and (**c**) GBP/PEO filament.

**Figure 4:**
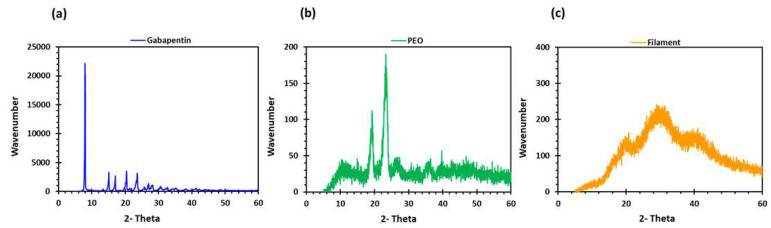
XRD profiles of (**a**) gabapentin; (**b**) polyethylene oxide (PEO); and (**c**) GBP/PEO filament.

PEO filaments loaded with gabapentin were successfully extruded using HME without any plasticiser (Figure 1). A drug loading of 98% efficiency was achieved in the extruded filaments and was within the pharmacopeial assay limit (Table 1). Thermal analyses (DSC and TGA) were conducted on the powdered samples and the extruded filaments. The DSC of the pure gabapentin powder showed that it has a crystalline structure with a sharp endothermic melting peak at approximately around 170 °C, Figure 2a. However, two thermal events were observed during the DSC analysis of PEO, i.e., firstly, a sharp endothermic melting peak at approximately 65 °C which shows its crystalline nature; and secondly, an exothermic peak at approximately 160 °C which depicts the polymer decomposition [47] (Figure 2b). DSC thermogram of the extruded filament was also carried out, and no drug crystallinity or drug–polymer interaction was observed in the extruded filament (Figure 2c). The TGA profiles showed that the onset of degradation for the drug and polymer was above the operating temperature (115 °C) used in the printing process (Figure 3). Interestingly, the thermal data (DSC and TGA) of the HME extruded filament showed greater stability as compared to those of GBP and PEO, as there has been no degradation peak. This behaviour may be attributed to the plasticisation of the polymer by the GBP which has a low comparatively molar mass. This may have resulted in the reduction in secondary forces (hydrogen bonding and van der Waals forces) between the GBP and PEO polymeric chains by occupying the intermolecular spaces. Hence, this leads to the alteration in the three-dimensional organisation which resulted in the greater thermal stability of the extruded filaments [48]. In addition to DSC and TGA, an XRD analysis was also carried out that confirmed the crystalline nature of gabapentin and PEO as sharp high-intensity peaks were observed (Figure 4a,b). Moreover, no high-intensity peaks of gabapentin and PEO were observed in the extruded filaments, which indicates the successful dispersion of the drug in the polymer (Figure 4c). In summary, it was concluded that the DSC and XRD results coincided with each other, as both analyses confirmed the crystalline nature of gabapentin and PEO, whereas the extruded drug-loaded filaments were of amorphous nature.

Filaments should possess good mechanical properties to be used for 3D printing. If the filament is too brittle, it will break while feeding through the gears of the FDM printer and if the filament is too soft, it will collect inside instead of extruding through the nozzle [49,50]. A three-point bending test was, therefore, performed to investigate the mechanical properties of the filaments. The force and the stress required to break the filaments were found to be 2.4 N and 17.22 MPa, respectively. The breaking distance for the filament was 5.9 mm, whereas Young’s modulus was 18.12 MPa (Table 1). The breaking distance which shows a toughness of the filament should not be less than 1.5 mm [46]. Moreover, the successful extrusion of PEO filaments without any plasticiser can be attributed to the basic structural unit of the PEO polymer which is the ethylene glycol skeleton [HO-(CH_2_-CH_2_-O)n-H]. It is basically the molecular weight of the polymer that defines the grades of PEO. Below the molecular weight of 25,000 Da, PEOs are known as polyethylene glycols (PEGs) and PEGs are widely utilised due to their non-toxicity and biodegradability [51,52]. Because the structural unit in PEO and PEG is the same, it was possible to extrude the PEO filaments without any plasticiser. The literature has also reported the extrusion of PEO at temperatures higher than its melting point and without the aid of any plasticisers [53]. Overall, the filaments possessed good mechanical properties and after characterisation, the filaments were successfully used to print the matrix tablets with varying shell number and infill density (Figure 1b–h).

### 2.2. Development and Characterisation of 3D-Printed Matrix Tablets

The extruded filaments were successful in printing the tablets. Seven different types of tablet formulations with the same formulation composition (PEO:Gabapentin—80:20%) and dimensions (7 × 4 mm) but varying numbers of shells (1, 2, 3, and 4) and infill densities (10%, 20% and 30%) were printed (Table 2).

**Figure 5:**
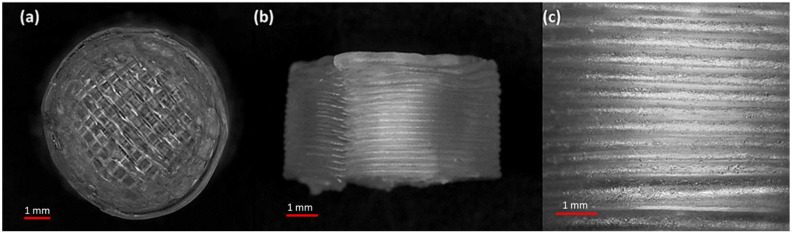
SEM micrographs of a 3D-printed tablet, namely F2 (2 shells with 0% infill): (**a**) whole matrix tablet; (**b**) side view of the matrix tablet; and (**c**) the side surface view.

Table 3 shows that the drug loading in the 3D-printed tablets was within the pharmacopeial assay limit. Moreover, the statistical findings show that the drug-loading differences among the various formulations (F1–F7) were not statistically significant, *F_stat_ < F_crit_* and *p* > 0.05. The breaking strengths of the tablets were also measured, and the values were in the range of 398.66–421.55 N. These values show that the printed tablets were robust enough to withstand rough handling and the friability of all the printed tablets was also 0%. The SEM image analysis of the printed tablets (Figure 5) shows the layer-by-layer manner of the printing technique. Although the surface of the tablet is not smooth, the layering pattern, which is visible on the side view (Figure 5b,c), confirms the successful extrusion of filaments with good mechanical properties.

**Figure 6:**
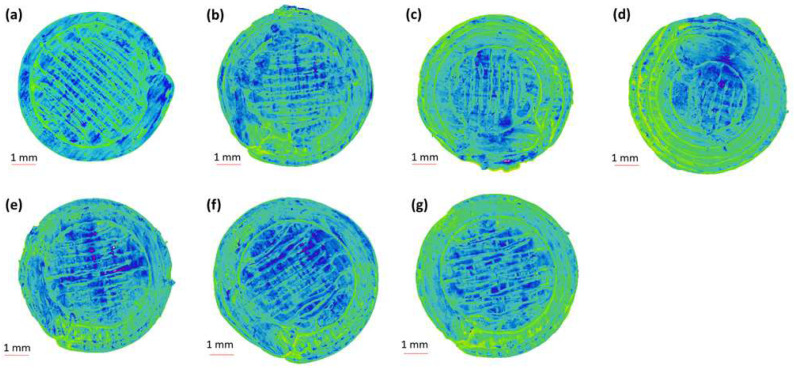
Two-dimensional surface texture images of 3D-printed tablet: (**a**) F1 (1 shell with 0% infill); (**b**) F2 (2 shells with 0% infill); (**c**) F3 (3 shells with 0% infill); (**d**) F4 (4 shells with 0% infill); (**e**) F5 (2 shells with 10% infill); (**f**) F6 (2 shells with 20% infill); and (**g**) F7 (2 shells with 30% infill).

**Figure 7:**
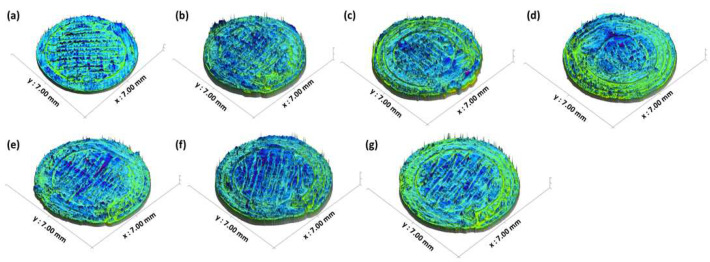
Three-dimensional surface texture images of 3D-printed tablet: (**a**) F1 (1 shell with 0% infill); (**b**) F2 (2 shells with 0% infill); (**c**) F3 (3 shells with 0% infill); (**d**) F4 (4 shells with 0% infill); (**e**) F5 (2 shells with 10% infill); (**f**) F6 (2 shells with 20% infill); and (**g**) F7 (2 shells with 30% infill).

**Figure 8:**
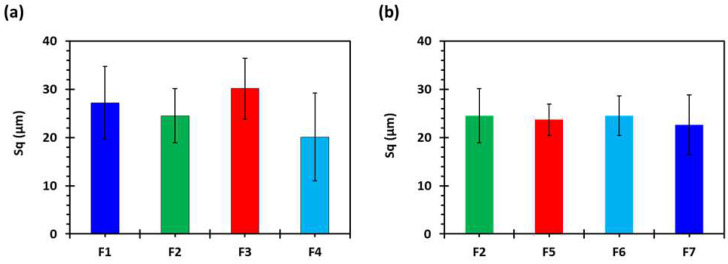
Surface roughness properties of 3D-printed tablets, showing the effect of (**a**) shell number and (**b**) infill percentage (*n* = 5). F1 (1 shell with 0% infill); F2 (2 shells with 0% infill); F3 (3 shells with 0% infill); F4 (4 shells with 0% infill); F5 (2 shells with 10% infill); F6 (2 shells with 20% infill); and F7 (2 shells with 30% infill).

The 2D and 3D surface texture analyses were performed using an ETL-based imaging system, as shown in the Figure 6 and Figure 7. The layering pattern of the tablets was evident in the 2D images (Figure 6) and showed an almost identical surface texture for all the tablets. In addition, the varying shell numbers in formulations F1–F4 are also evident in Figure 6a–d. Three-dimensional images, on the other hand, showed the presence of the inordinate peaks and the valleys on the surface of the printed tablets (Figure 7). To further explore the surface texture analysis, surface texture parameter, root mean square roughness (Sq), was also studied. Figure 8 shows the Sq values of all the 3D-printed tablets and it is evident that there was no significant difference in the Sq values of the 3D-printed tablets. This is because the varying parameters, i.e., the shell number and infill density, during the printing, result in changes on the inside and not on the surface of the tablet [54]. Therefore, the Sq values of all the formulations are close to each other and are not statistically different.

### 2.3. Ex Vivo Mucoadhesion Studies 

Mucoadhesion is the interaction between the pharmaceutical dosage form and the mucus membranes enabling a better contact between the formulations and membranes to prolong the residence time [55]. The process involves the wetting and hydration of the polymer, which is known as the contact stage and is essential in developing the mucoadhesive interaction. The next stage consists of interpenetration between mucosal membrane and polymeric chains, forming a chemical bond due to the hydrogen bonding, van der Waals, or electrostatic interaction [56]. These surface forces result in adhering the substance to the mucosal membrane. PEO consists of long and linear chains of ethylene oxide. At a low molecular weight, PEO possesses very weak mucoadhesive properties, i.e., forms weaker gels with mucus, which, may be, is due to the less available sites for hydrogen bonding or conformation for interpenetration between polymeric chains and the mucosal membrane is not favourable. Therefore, high concentrations of low-molecular-weight PEO are required to detect any mucoadhesion. However, on the other hand, PEO with high molecular weight possesses excellent mucoadhesive properties and does not require high concentrations [57].

**Figure 9:**
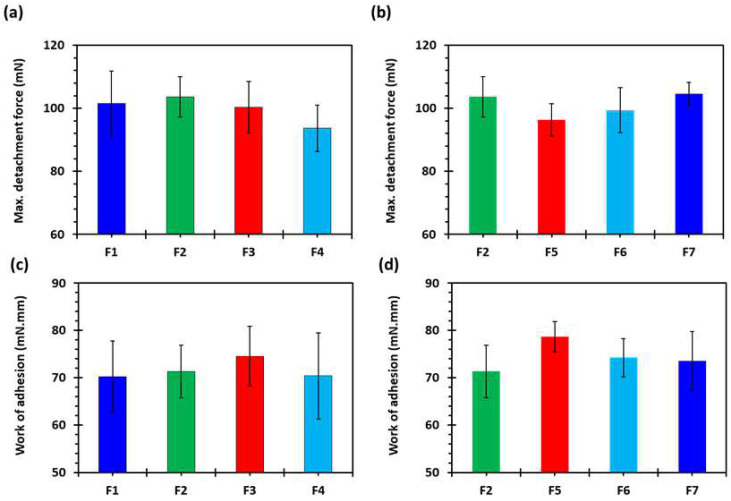
(**a**,**b**) Maximum mucoadhesion detachment force and (**c**,**d**) work of adhesion against porcine stomach mucosa in 0.1 N HCl at 37 ± 0.5 °C (*n* = 3): F1 (1 shell with 0% infill); F2 (2 shells with 0% infill); F3 (3 shells with 0% infill); F4 (4 shells with 0% infill); F5 (2 shells with 10% infill); F6 (2 shells with 20% infill); and F7 (2 shells with 30% infill).

Figure 9a,b shows the maximum mucoadhesion detachment force and Figure 9c,d shows the work of adhesion against a porcine stomach mucosa in 0.1 N HCl solution at 37 ± 0.5 °C. It is evident from Figure 9a,b that the maximum detachment force was high for all the formulations. The values were not statistically different, however, the F2 formulation showed the highest detachment force and F4 showed the lowest. There was also no difference in the work of adhesion (Figure 9c,d). The reason for there not being markedly different behaviour is that the mucoadhesion phenomenon involves contact between the surfaces, whereas all the tablet formulations with a varying shell number and infill density are different from the inside and there is no change on the surface [54].

### 2.4. In Vitro Floating and Drug Release Studies 

**Figure 10:**
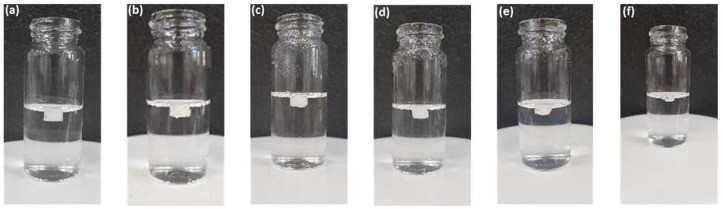
Images of 3D-printed tablet; F2 (2 shells with 0% infill) floating in dissolution medium (0.1 N HCl solution) at 37 ± 0.5 °C (**a**) t = 2 h; (**b**) t = 4 h; (**c**) t = 6 h; (**d**) t = 8 h; (**e**) t = 10 h; and (**f**) t = 12 h.

The major reason for printing the tablets with a varying shell number and infill density was to investigate the floating capacity so that the tablet can stay in the stomach for longer periods and provide extended drug release. Figure 10 shows the images of floating F2 tablets at different time intervals. During the dissolution test, the F2 formulation with a density of 0.77 mg/mm^3^ (2 shells, 0% infill) produced the highest floating ability, i.e., floated for more than 10 h, followed by F1 (1 shell, 0% infill) with a density of 0.73 mg/mm^3^ and a floating time of less than 8 h. The F4 tablets (4 shells, 0% infill) were the densest (0.93 mg/mm^3^) followed by F7 (2 shells, 30% infill) with a density of 0.89 mg/mm^3^. Both formulations floated for less than 2 h and tended to sink to the bottom of the vessel (Table 4). Overall, it was concluded that the tablet density increased with the increase in the shell number and infill density; however, the floating time did not follow the trend and decreased with an increase in tablet density. The tablets with a density above 0.84 mg/mm^3^ did not float for long periods and sank in less than 2 h (Table 4).

**Figure 11:**
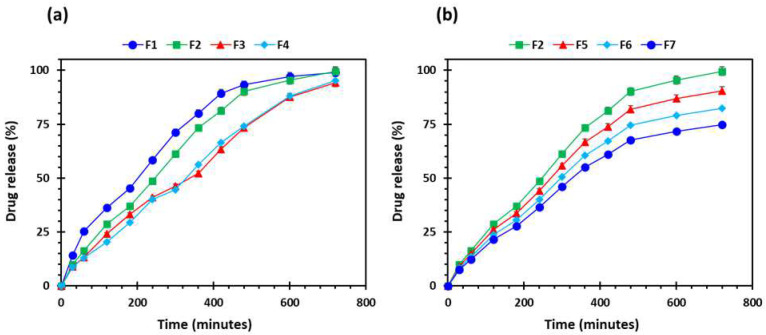
Drug release profiles of the 3D-printed tablets (*n* = 5): (**a**) the effect of number of shells; and (**b**) influence of percentage infill: F1 (1 shell with 0% infill); F2 (2 shells with 0% infill); F3 (3 shells with 0% infill); F4 (4 shells with 0% infill); F5 (2 shells with 10% infill); F6 (2 shells with 20% infill); and F7 (2 shells with 30% infill).

PEO is a linear, uncross-linked, and a hydrophilic polymer which, once in contact with dissolution media, hydrates and begins the disentanglement of polymeric chains. The persistent penetration of dissolution media and interaction between polymeric chains and penetrating media results in accommodating water molecules, which leads to the formation of a layer of hydrogel around the matrix tablet. Hydrogel, a semi-dilute or concentrated solution of the polymer, outside the dry core, is basically formed as PEO reaches its glass transition temperature (Tg), the temperature at which glass-to-rubbery phase transition occurs. The Tg of a polymer is an important characteristic to know with respect to a sustained release drug delivery system as the polymeric chains are not mobile below the Tg. In comparison, the polymeric chains are highly mobile above the Tg. The drug release rate is controlled by the hydration and dissolution of the polymer, which depends on the viscosity and concentration of the polymer [58,59]. In this study, we kept the viscosity and concentration of the polymer constant. Figure 11 shows the drug release from all the printed formulations. All the formulations showed the sustained release of the drug. Figure 11a shows the effect of varying shell numbers by keeping the infill density constant on the drug release from the printed tablets. It can be seen from Figure 11a that F1 showed a faster drug release followed by F2. Drug release from F3 and F4 were both slow and the release decreased with an increase in the shell number. The same trend was seen with respect to the increase in the infill density, i.e., the drug release decreased with the increase in the infill density. Figure 11b shows the gradual release of the drug from the tablets; nonetheless, the F2 tablets showed the faster drug release and F7 showed the slowest drug release. This is because by increasing the shell number and infill density, we are increasing the amount of filament (drug and polymer) in the tablet [54]. In addition, at low infill density there is less deposition of the material which results in the formation of more porous tablet leading to faster release of the drug. On the contrary, increase in infill density will increase deposition of the material which will result in less porous tablet with slow release of the drug [60]. Among all the formulation, the F2 formulation was chosen for in vivo studies as it showed the highest floating capacity with sustained release of the drug.

### 2.5. Pharmacokinetic (In Vivo Drug Absorption) Studies 

**Figure 12:**
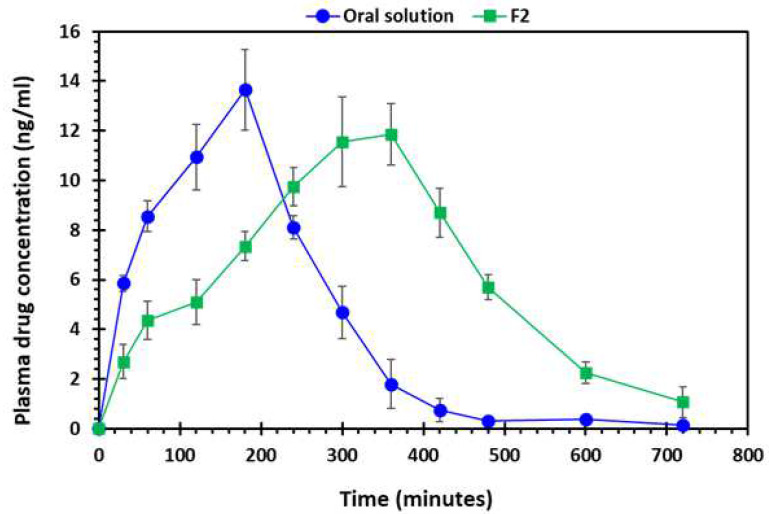
Plasma drug (gabapentin) absorption vs. time profile of oral solution and 3D-printed tablet, F2 (2 shells with 0% infill), *n* = 6.

Figure 12 shows the drug absorption versus time profile of the oral solution and 3D-printed tablet. It is evident from the findings that the pharmacokinetic parameters of the oral solution and F2 tablet were markedly different from each other (Table 5). In addition, the AUC of the F2 printed tablets was significantly higher (*p* < 0.05) than the oral solution. The maximum plasma concentration (C_max_) was higher for the oral solution and lower for the printed tablet, however, the difference was statistically not significant (*p* > 0.05). However, the time required to achieve C_max_ known as T_max_ was distinctly longer for the F2 tablet compared to the oral solution. This is because the 3D-printed tablets contain PEO and the extended-release enabled the drug to be absorbed into bloodstream over a longer period compared to the oral solution.

## 3. Materials and Methods

### 3.1. Materials

Gabapentin was purchased from TCI Europe (Zwijndrecht, Belgium). Polyethylene oxide (PEO), a carrier polymer, with an average viscosity molecular weight of 200,000 (Mw) was purchased from Sigma-Aldrich Ltd., Old Brickyard, New Rd, Gillingham, UK. All other chemical reagents used were of analytical grade and used as supplied.

### 3.2. Preparation of Filaments 

A single screw extruder (Noztek^®^ Pro pellet and powder extruder, Sussex, UK) was employed for the extrusion of gabapentin-loaded filaments using a powder blend of PEO:Gabapentin. The HME parameters used during the extrusion of filaments are given in Table 6. The nozzle diameter and extrusion temperature were 1.75 mm and 105 °C, respectively. Once successfully extruded, the filaments were stored in a desiccator at room temperature until further investigation.

### 3.3. Physicochemical Characterisation of Filaments

#### 3.3.1. Determination of Drug Loading 

A gabapentin-loaded filament (0.2 g) was placed in a 1 L water: methanol (1:1) solvent mixture, under magnetic stirring until complete dissolution. Jenway 6405 UV spectrophotometer, Staffordshire, UK, was then used to analyse the filtered liquid samples to determine the gabapentin content. All the measurements were carried out in triplicate. 

#### 3.3.2. Differential Scanning Calorimetry (DSC)

The DSC of all the powder samples and extruded filaments was carried out a using Mettler Toledo SC 821, Mettler-Toledo Ltd., Leicester, UK. Briefly, standard aluminium pans were used to place 5–10 mg of samples to run the analysis under a nitrogen flow of 50 mL/min and temperature program of 10 °C/min from 25–300 °C.

#### 3.3.3. Thermogravimetric Analysis (TGA)

TGA was performed using a Mettler Thermobalance TG50 (Mettler-Toledo Ltd., Leicester, UK). All the powder samples, plain and blended, and drug-loaded filaments were placed in open aluminium crucibles for analysis. The samples were heated from 25–300 °C at heating rate of 10 °C/min. Nitrogen gas was used as a purge gas with a flow rate of 50 mL/min. 

#### 3.3.4. X-ray Diffraction Studies (XRD)

D2-Phase X-ray diffractometer (Bruker UK Ltd., Coventry, UK) equipped with a CuKα radiation source at 30 KV voltage and 10 mA current was used for the XRD study of all the powder samples (polymer, drug, powder blend). The 2-theta (θ) range of 5°–100° using 0.02 step size settings was used to obtain the diffraction patterns.

#### 3.3.5. Scanning Electron Microscopy (SEM)

SEM was used to examine the morphology of drug-loaded filaments. Briefly, double-sided adhesive tape was used to mount the samples onto stubs. A Quorum SC7620 Sputter Coater (Quorum Technologies, Laughton, UK) was then used for the sputter-coating of samples with palladium/gold (20:80) for 60 s and were photometrically examined Jeol JSM-6060CV, Jeol Inc., Peabody, MA, USA [61].

#### 3.3.6. Mechanical Testing of Filaments

The TA-XT2i texture analyser (Stable Micro Systems Ltd., Surrey, UK) was used to carry out mechanical testing. All the extruded filaments were cut into 10 cm pieces and placed onto TA-95N 3-point bend probe set which was attached to the analyser, maintaining the moving speed of the blade at 5 mm/s until it reached 15 mm under the sample. Exponent^®^ software (Stable Micro Systems Ltd., Surrey, UK) was then used to analyse the collected data. 

#### 3.3.7. Fabrication of 3D Printed Tablets

**Figure 13:**
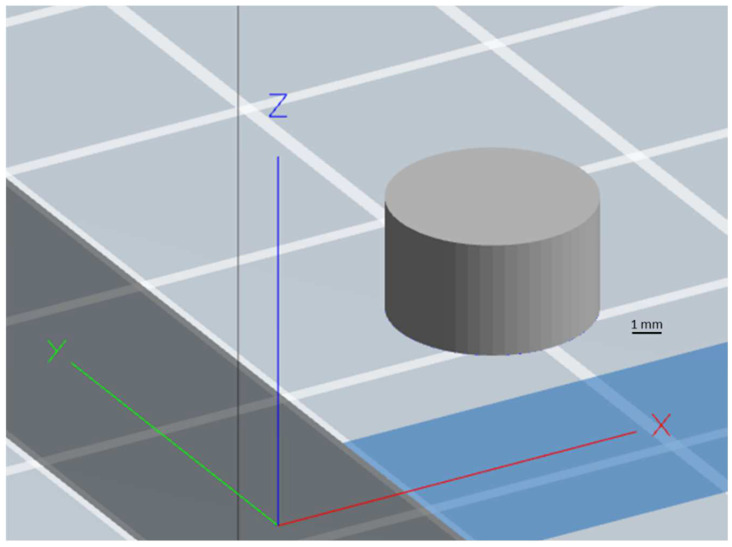
CAD design of the cylindrical tablet developed using SolidWorks^®^ version 2015.

**Figure 14:**
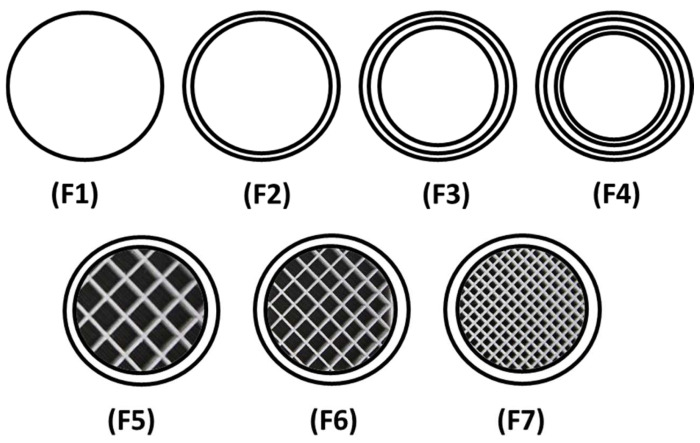
Schematic illustration depicting the cross-sectional view of 3D-printed matrix tablets showing the infill patterns and shells: F1 (1 shell with 0% infill); F2 (2 shells with 0% infill); F3 (3 shells with 0% infill); F4 (4 shells with 0% infill); F5 (2 shells with 10% infill); F6 (2 shells with 20% infill); and F7 (2 shells with 30% infill).

A cylindrical tablet of 7 mm in diameter and 4 mm in height was designed using SolidWorks^®^ version 2015 (Figure 13), and the file was then converted into stl. (Stereolithographic) format for further use. Gabapentin-loaded filaments were loaded into MakerBot replicator mini (MakerBot^®^ Inc., New York, NY, USA) separately to print the tablets. Keeping the dimensions, infill pattern (line), top and bottom layer thickness constant, gabapentin tablets were printed with different shell numbers’ (1, 2, 3, and 4) infill density (10%, 20%, and 30%), respectively, as illustrated in Figure 14. All these parameters were digitally controlled using the Makerbot^®^ software (MakerBot^®^ Inc., New York, NY, USA). Other 3D printing parameters were kept constant including printing temperature (115 °C), height layer (0.1 mm), extrusion speed (90 mm/s), and travelling speed (150 mm/s).

### 3.4. Characterisation of 3D Printed Matrix Tablets

#### 3.4.1. Geometrical and Morphological Assessment of Matrices

To determine the height (*h*) and diameter (*d*) of the tablets, a digital Vernier calliper was used. The tablet height (*h*), radius (*r*) and the circular constant (*π*) were used to calculate the tablet volume (*v*), as shown in Equation (1). Moreover, the tablet mass (*m*) and volume (*v*) were then used to determine the tablet density (*ρ*), as can be shown in Equation (2) [62]. The surface morphology of the printed tablets was observed using SEM.

(1) *v* = π (*d*/2)^2^ × *h*

where *v* = volume of the tablet, *π* = circular constant, and *d* = diameter of the tablet

(2) $$\rho =\frac{m}{v}$$

where *ρ* = density of the tablet, *m* = mass of the tablet, and *v* = volume of the tablet

#### 3.4.2. Three-Dimensional Surface Texture Analysis 

**Figure 15:**
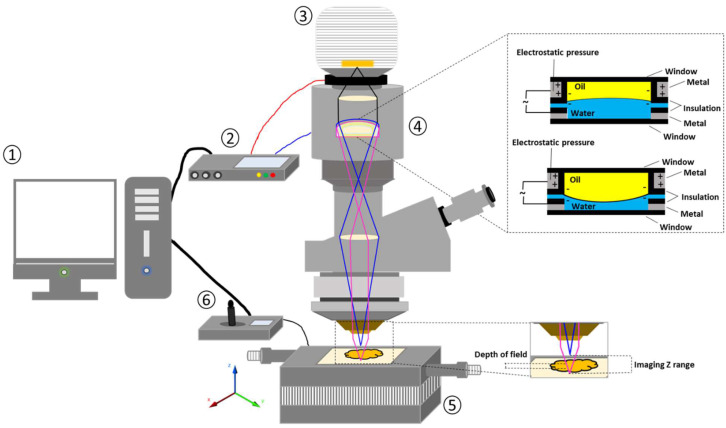
Schematic illustration of electrically tuneable lens (ETL)-based variable focus imaging system used for the surface texture analysis of the 3D-printed tablets: (1) computer; (2) ETL driver; (3) camera; (4) ETL; (5) automatic motorised vertical translation stage; and (6) stage controlling system [63].

A 3D surface texture analysis was carried out using an electrically tuneable lens-based imaging system (Figure 15) described and used by Nirwan et al., 2022 [63].The sample was prepared using a method described elsewhere [48,64].The tablet was placed on a stainless-steel wafer (3 × 3 cm) using double-sided adhesive tape and a whole tablet was scanned instead of a specified region. Afterwards, a 3D surface texture parameter, namely root mean square roughness (Sq), was calculated using software MATLAB^®^ 2017 software (The MathWorks, Inc., Natick, MA, USA). 

#### 3.4.3. Determination of Tablet Strength 

To measure the tablet strength, a Testometric M500–50 CT, Testometric Company Ltd., Rochdale, United Kingdom, machine was used. Ten tablets of each formulation were randomly selected and tested. The tablets were placed diametrically, and force was applied at the rate of 10 mm/min by moving the upper punch until the tablet was broken. 

#### 3.4.4. Determination of Tablet Friability

Ten tablets were randomly selected for friability testing. The tablets were weighed and placed in the tablet friability testing instrument (PTF 20E, Pharmatest, Hainburg, Germany), and the drum was rotated at a speed of 20 rpm for 5 min. After that, the tablets were weighed again to calculate the friability in terms of weight loss and expressed as a percentage of the original weight of the tablet. 

#### 3.4.5. Ex Vivo Mucoadhesive Studies

**Figure 16:**
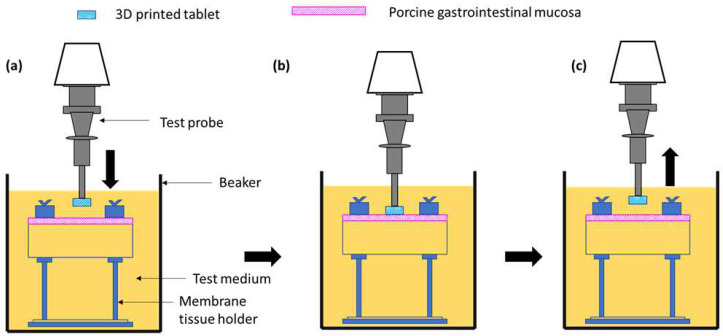
Schematic illustration of the mucoadhesive testing of 3D-printed tablets using the texture analyser with a specialised mucoadhesive holder: (**a**) probe attached with 3D-printed tablet was moved downward; (**b**) 3D-printed tablet was attached with gastrointestinal mucosa with specified force and time; and (**c**) probe was moved upward at a specified rate.

**Figure 17:**
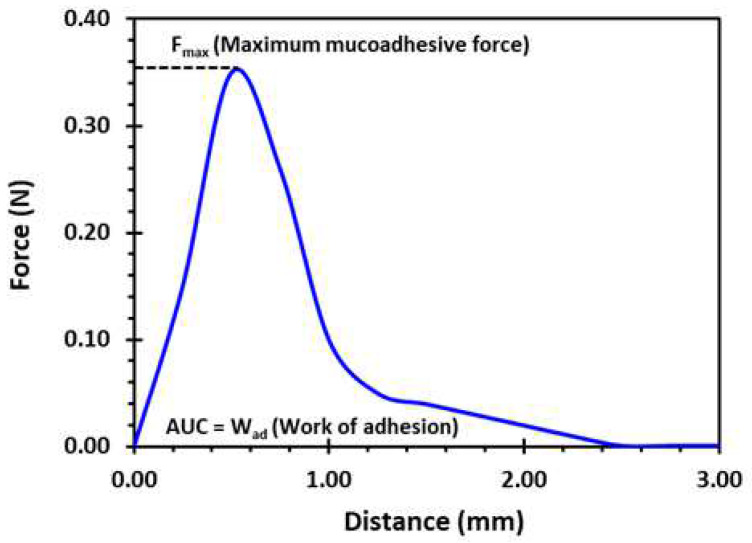
A typical force vs. distance profile for a 3D-printed tablet (F2, (2 shells with 0% infill)) from the mucoadhesion test performed in 0.1 N HCl (150 mL) at 37 °C using the texture analyser. Fmax is the highest force required to separate the probe from the gastric tissue which is determined from the maximum peak height of the profile. Wad is the total force involved in the probe separation during withdrawal phase which is calculated from the area under the curve (AUC) of the force vs. distance profile.

A texture analyser equipped with a mucoadhesive holder was used to study the mucoadhesive property of the formulated matrix tablets [65]. The 3D-printed tablet was attached to the cylindrical probe having 10 mm diameter using double-sided tape. Porcine gastrointestinal mucosa (20 × 20 mm) was equilibrated at 37 ± 0.5 °C for 15 min before being placed on the stage of the mucoadhesive holder and the temperature of 37 °C was then maintained in 150 mL of 0.1 N HCl. Figure 16 exhibits the schematic illustration of the mucoadhesive testing of 3D-printed tablets using the texture analyser with specialised mucoadhesive holder: (a) probe attached with a 3D-printed tablet was moved downward; (b) a 3D-printed tablet was attached with gastrointestinal mucosa with specified force and time; and (c) a probe was moved upward at a specified rate. The specified force applied was 0.5 N and the contact time between the tablet and the gastrointestinal mucosa was 200 s. The probe was withdrawn at a speed of 0.2 mm/s. Software (Exponent Connect, Stable Micro Systems Ltd., Surrey, UK) was used to measure the maximum detachment force, Fmax, required to separate the probe from the gastrointestinal mucosa. The work of adhesion (Wad), which is the total force involved in the probe separation during the withdrawal phase, was also calculated from the area under the curve (AUC) of the force vs. the distance profile (Figure 17).

#### 3.4.6. In Vitro Dissolution Testing

Dissolution was studied using USP II paddle apparatus where the temperature and paddle rotation speed were maintained at 37 °C and 50 rpm, respectively. Tablets were placed in 900 mL simulated gastric fluid media (pH 1.2). The total time of the dissolution study was 12 h. Five-millilitre aliquots were drawn from each vessel at predetermined interval times and replenished with 5 mL of dissolution medium. HPLC was utilised to analyse the dissolution samples with an injection volume of 20 µL. Acetonitrile:ammonium carbonate (5:95) at pH 8.0 was used as the mobile phase for gabapentin at the flow rate of 1 mL/min and gabapentin was detected at a wavelength of 340 nm via UV-detection. All the measurements were carried out in triplicate.

#### 3.4.7. Floating Test 

An in vitro floating test was also carried out along with the dissolution test. The gastroretentive gabapentin tablets were transferred from the dissolution vessels to 15 mL glass vials containing the dissolution medium at different time intervals (2 h, 4 h, 6 h, 8 h, 10 h, and 12 h) and the photos were then taken immediately. The tablets were returned to the dissolution vessel after taking the photos. 

#### 3.4.8. Pharmacokinetic Studies

The in vivo study was carried out on white albino rabbits weighing 2.8–3.5 kg. The rabbits were further categorised into two groups, each containing 6 rabbits per group. All rabbits were housed individually in cages and under environmentally controlled conditions (25 ± 1 °C; 44 ± 3% relative humidity). Rabbits were given free access to water and food until the last 24 h before the experiment was started. After this, the rabbits had free access to water but not the food. The study protocol was approved by the Pharmacy Research Ethics Committee (PREC) at the University of Sargodha, Sargodha, Pakistan (UOS/PERC/102).

A single-dose pharmacokinetic study was carried out in which the rabbits in group 1 were administered an oral solution of gabapentin (25 mg in 0.5% methylcellulose-based oral solution) and 3D-printed gabapentin tablets (F2 containing 25 mg gabapentin) were administered to the group 2 [66,67]. At different time intervals (0, 30, 60, 120, 240, 300, 360, 420, 480, 600, and 720 min), 1 mL of blood samples was collected from the marginal ear vein into heparinised tubes. The collected blood samples were then centrifuged for 10 min at an ambient temperature. After centrifugation, the plasma layer was separated and stored at –20 °C until analysed.

Fifty-microlitres of the rabbit plasma was added to amlodipine besylate solution and 100 µL of acetonitrile was also added for protein precipitation and centrifuged for 10 min. The supernatant layer was separated in a clean tube and 1 mL of acetonitrile, 30 µL of 1-fluoro-2,4-dinitrobenzene (FDNB, derivatisation agent), and 200 µL of 0.25 M borate buffer were added. The tube was then inverted and kept at 65 °C for 10 min to allow a derivatisation reaction. After derivatisation, the samples were brought to room temperature and 25 µL of the 1 M HCl solution was added, before the samples were inverted and dried. Once dried, the residues were mixed with 200 µL of mobile phase and 20 µL were injected into an HPLC containing reverse phase C-18 column (Phenomenex). The mobile phase used was 50 mM sodium phosphate buffer (pH 3.9): methanol (27:73, *v*/*v*), at a flow rate of 1.2 mL/min and UV wavelength of 360 nm was used to detect the gabapentin [67]. The PKSolver program, an add-in macro for Microsoft Excel^®^, was employed for the calculation of the different pharmacokinetic parameters.

#### 3.4.9. Statistical Analysis

Analysis of variance (ANOVA) (confidence limit of *p* < 0.05) was used to investigate the statistical significance of drug loading and pharmacokinetic parameters. 

## 4. Conclusions

The present study successfully addressed the absorption and personalisation issues of gabapentin by developing 3D-printed gastro retentive floating matrix tablets. The present study reports the successful extrusion of PEO filaments loaded with gabapentin without using any plasticiser and was successfully employed in developing the printed matrix tablets with varying shell numbers and infill densities. The matrices showed different floating capacities, and it is concluded that an increase in infill density and shell number did not increase the floating time. All the tablets showed extended drug release; however, the extent of drug release decreased as the shell number and infill density increased. Moreover, the current report has shown that the floating time and drug release can be modified by altering the 3D printing parameters. Overall, it can be concluded that 3D printing technology is an easy-to-use approach which demonstrated its benefits in developing medicines based on a mucoadhesive gastro-retentive strategy, improving the drug absorption of gabapentin and its capacity to effectively manage OAB.

## Figures and Tables

**Table 1:** Drug loading and three-point bending results of filaments (*n* = 10, standard deviations are in parenthesis).

Drug Loading (%)	Force (N)	Distance (mm)	Stress (MPa)	Strain	Young Modulus (E) MPa
98.5 (1.05)	2.4 (0.22)	5.9 (1.15)	17.22 (1.35)	0.95 (0.21)	18.12 (1.19)

**Table 2:** Formulation composition, dimension, and printing parameters of tablets.

Formulation Code	Formulation Composition (Weight Ratio %)	Dimensions (D × H mm)	Shell Number	Infill Percentage (%)
F1	PEO:Gabapentin (80:20)	7 × 4	1	0
F2	PEO:Gabapentin (80:20)	7 × 4	2	0
F3	PEO:Gabapentin (80:20)	7 × 4	3	0
F4	PEO:Gabapentin (80:20)	7 × 4	4	0
F5	PEO:Gabapentin (80:20)	7 × 4	2	10
F6	PEO:Gabapentin (80:20)	7 × 4	2	20
F7	PEO:Gabapentin (80:20)	7 × 4	2	30

**Table 3:** Drug loading and mechanical properties of 3D-printed tablets.

Formulation Code	Drug Loading (%)	Breaking Strength of Tablets (N)	Friability (%)
F1	97.51 (0.75)	411.65 (5.59)	0
F2	98.33 (1.10)	404.32 (6.20)	0
F3	100.05 (2.53)	399.21 (11.39)	0
F4	99.1 (2.65)	421.55 (10.35)	0
F5	97.33 (0.61)	398.66 (12.35)	0
F6	98.21 (1.10)	414.99 (15.36)	0
F7	98.87 (0.56)	410.36 (6.55)	0

**Table 4:** Physical parameters and floating time of 3D-printed tablets (*n* = 5).

Formulation Code	Measured Volume (mm^3^)	Measured Mass (mg)	Tablet Density (mg/mm^3^)	Floating Time (h)
F1	150.69 (0.98)	110.15 (1.21)	0.73 (0.01)	>8
F2	154.32 (1.10)	120.15 (2.42)	0.77 (0.02)	>10
F3	157.16 (3.15)	130.82 (3.73)	0.83 (0.03)	>6
F4	154.12 (3.11)	144.28 (2.25)	0.93 (0.01)	<2
F5	149.49 (2.82)	124.3 (3.36)	0.83 (0.02)	>6
F6	160.50 (4.28)	135.62 (4.11)	0.84 (0.02)	>6
F7	158.63 (3.92)	141.21 (3.51)	0.89 (0.04)	<2

**Table 5:** Pharmacokinetic parameters of gabapentin solution and 3D-printed tablet (F2, 2 shells with 0% infill) after oral administration, *n* = 6, n.c.: not computable.

Parameters	Oral Solution	F2	*p*-Value
T_1/2_ (min)	79.99 (6.22)	97.96 (8.64)	0.04
T _max_ (min)	180 (0.00)	360 (0.00)	n.c.
C _max_ (ng/mL)	13.65 (3.21)	11.85 (2.29)	0.32
AUC _0–t_ (ng/mL/h)	3037.05 (174.81)	4381.65 (251.37)	0.007

**Table 6:** HME parameters for developing filaments.

Formulation (Weight Ratio)	Extrusion Temperature (°C)	Screw Speed (rpm)	Torque (N/cm)
PEO:Gabapentin (80:20)	105	30	18

## Data Availability

Data is contained within the article.
